# Drastic sex-dependent etiological distribution in severe liver diseases from Gabon

**DOI:** 10.3389/fonc.2022.907554

**Published:** 2022-09-15

**Authors:** Pamela Moussavou-Boundzanga, Patrice Emery Itoudi Bignoumba, Augustin Mouinga-Ondeme, Berthe Amelie Iroungou, Berthold Bivigou-Mboumba, Agnès Marchio, Maryam Saibou, Jean-Baptiste Moussavou Kombila, Pascal Pineau

**Affiliations:** ^1^ Laboratoire de Biologie Moléculaire et Cellulaire (LABMC), Université des Sciences et Techniques de Masuku (USTM), Franceville, Gabon; ^2^ Unité des Infections Rétrovirales et Pathologies Associées, Centre International de Recherches Médicales de Franceville (CIRMF), Franceville, Gabon; ^3^ Service de Gastroentérologie, Centre Hospitalier Universitaire de Libreville (CHUL), Libreville, Gabon; ^4^ Unité Mixte de Recherche Centre International de Recherches Médicales de Franceville et Service de Santeí Militaire (CIRMF-SSM), Libreville, Gabon; ^5^ Unité « Organisation nucléaire et oncogenèse », INSERM U993, Institut Pasteur, Paris, France

**Keywords:** middle africa, gabon, liver cirrhosis, hepatocellular carcinoma, primary liver cancer (PLC), hepatitis virus, alcohol, sex-difference

## Abstract

Chronic liver diseases still represent a worrying public health issue in Sub-Saharan Africa. In this region, emphasis is generally made on hepatocellular carcinoma (HCC) albeit liver cirrhosis (LC) is also responsible for an important death toll. Very few studies have compared the presentation and etiologies of cancer and cirrhosis of the liver in Middle Africa. We conducted a comparative retrospective analysis of 74 and 134 cases of patients with HCC and LC treated in Libreville, Gabon. Viral or lifestyle risk factors, clinical symptoms, and biological features were compared. We observed that ages of diagnosis were 53.2 ± 15.7 years and 48.6 ± 18.6 years for HCC and LC with remarkably low M:F sex ratios (1.3–1.8). Ethanol consumption was highly prevalent in both disease types (65.0%–70.0%). Chronic viral infections with hepatitis B (HBV) or C (HCV) virus were also widespread with slight domination of the former in both diseases (43.4% vs. 34.3%, and 35.9% vs. 28.5%). Patients with HCC were presenting very late with a mean diameter of the main nodule of 84 ± 50 mm and a multifocal pattern in 72.7% of cases. HCC developed on a cirrhotic liver in 91.7% of cases. Serum AFP was frankly elevated (>400 ng/ml) in only 35.8% of HCC cases. The most striking feature of the HCC series was the contrasted contribution of distinct pathogenic etiologies involving sex, viral, metabolic, and toxic factors. A frequently dysmetabolic condition synergizing with hepatitis C (anti-HCV, 73.8% vs 22.7%, p < 0.0001) in females and a male cancer promoted by recreational toxicants and chronic hepatitis B (HBsAg, 83.5% vs 35.9%, p < 0.0001) were observed. Men with HCC were considerably younger than women (46.8 ± 14.5 years vs. 62.2 ± 12.2 years, p < 0.0001). Further studies are now warranted to identify routes of HCV transmission and if they are still fueling reservoirs of future patients. Public policies to prevent alcohol-related harm have also to be urgently implemented in Gabon.

## Introduction

Chronic liver diseases represent a significant burden of morbidity and death in Sub-Saharan Africa albeit with substantial regional variations of incidence (1). Middle Africa is a vast territory that regroups nine nations and a population of 169 million habitants spread over 6.6 million km^2^. In this area, primary liver cancer incidence is generally considered as lower than in neighboring West Africa while the importance of liver cirrhosis (LC) is, overall, poorly known and subjected to a far lesser number of medical publications (2).

Gabon is a small country of two million habitants occupying a central position in Middle Africa. Gabon is characterized, according to some authors including our group, by a substantial underestimation of primary liver cancer or hepatocellular carcinoma (HCC) incidence (3). Nevertheless, infectious risk factors (RF) of HCC, such as chronic infection by hepatitis B and C viruses, are notoriously highly prevalent in the Gabonese population while alcohol consumption is known to be one of the highest in Sub-Saharan Africa (4). The role played by aflatoxin B1 on the epidemiology of terminal liver disease, often important in Africa, is currently unknown in Gabon just as the role of dysmetabolic conditions (obesity, type 2 diabetes, etc.) (5, 6).

Local epidemiology of HCC is well-known to be modulated by RF distribution and genetic architecture of the population. Besides, the descriptive epidemiology of any disease endemic to a given population is a prerequisite to implement the most appropriate preventative measures and to conduct an efficient screening of the persons at risk for complications. Unfortunately, due to significant international variations, extrapolation of observations made in a given population are not always transposable to another one (7, 8). There is currently no original paper that describes the presentation of HCC and LC in Gabon. In the current work, we explored, retrospectively, 74 patients with hepatocellular carcinoma (HCC) and 134 cases of LC who attended care in the Centre Hospitalier Universitaire de Libreville (CHUL) between 2011 and 2019.

## Patients and methods

### Patients

A series of 208 patients were retrospectively recruited in the Department of Internal Medicine from CHUL between 2011 and 2019. The criteria of inclusion for HCC were the presence of a liver mass above 1 cm in diameter at ultrasound or computed tomography with an arterial enhancement and a wash-out in venous phase, and/or a serum α-fetoprotein concentration above 400ng/ml in a clinical context of chronic liver disease (9, 10). LC was scored in case on the combination of portal hypertension, hepatocellular insufficiency, and changes in the hepatic parenchyma at ultrasound. No histological or elastometric appraisal of cirrhosis was available.

The etiological diagnosis of disease was attributed to HBV when HBsAg or HBV DNA was present; it was for HCV when anti-HCV immunoglobulins or HCV RNA were present. The causality of alcohol intoxication was retained when consumption was estimated to exceed 60 g/day. Clinical and biological data were collected at the time of admission to the department and before starting treatment. Most of the patients were unaware of their conditions and did not receive any antiviral therapy before the diagnosis of already severe disease.

The criteria for exclusion were the lack of clinical and/or biological data and the detection of liver nodules concomitant to an extra-hepatic primary tumor.

Due to the retrospective nature of the study, no informed consent was obtained for the patients before 2019, while patients recruited in 2019 gave their informed consent to participate in the study. The institutional review board approved the study. The study was conducted in accordance with the recommendations of the Convention of Helsinki.

### Statistical analyses

Statistical analysis was performed using the Prism 8.0.2 statistical package (GraphPad, USA) and, in case of very small p values (p < 1.0 E-04), with the BiostaTGV online software (https://biostatgv.sentiweb.fr/?module=tests) to improve the appraisal of significance by readers. Numerical variables were compared for analyses of two groups either by a Student’s *t*-test, by a Mann–Whitney *U* test as appropriate. When more than two groups were examined, ANOVA or Kruskal–Wallis test were used. Categorical variables were summarized as frequencies that were compared by Fisher’s exact test when two groups were compared or by χ^2^ in presence of more than two groups. All tests were univariate and two-sided. The level of significance was set at p < 0.05. Principal component analysis was performed as well with the Prism 9.4.0 statistical package.

## Results

### Patient demography

A total of 208 medical records with HCC or LC were enrolled at the Libreville University Hospital between 9 January 2011 and 23 July 2019. Demographical and clinical features are summarized in **Table 1**.

**Table 1:** Demographical and clinical features.

Clinico-biological features	HCC	LC	p-Value
	N = 74	N = 134	
*Demography*			
Sex ratio M :F	1.38 (43/21)	1.68 (84/50)	0.62 (ns)
Age (years)	53.2±15.7	48.6±18.6	0.0783 (ns)
*Risk Factors*			
HBsAg (%)	43.4	35.9	0.35 (ns)
Anti-HCV (%)	34.3	28.5	0.28 (ns)
Including B+C infections (%)	3.1	2.6	ns
nonBnonC (%)	25.7	37.0	ns
Anti-HIV1-2 (%)	14.5	5.1	0.0150
*Other risk factors*			
Ethanol consumption (%)	69.0	65.1	ns
Ethanol (g/24h)	105±97	97±77	0.66 (ns)
Tobacco consumption (%)	31.3	22.2	0.22 (ns)
Two Toxicants (Alcohol + Tobacco, %)	29.8	19.0	0.10 (ns)
Type 2 Diabetes (%)	17.1	8.1	0.0861 (ns)
Cryptogenicity (%)	6.7	7.7	ns
*Tumor features*			
Main diameter (mm)	84±50	na	
Multiple nodules (%)	72.7	na	
*Non-tumor liver status*			
Cirrhosis (%)	91.7	100.0	ns
Decompensation (%)	88.2	98.4	0.0033
Macronodular pattern (%)	66.6	45.2	0.0102
Heterogeneous echogenicity (%)	66.6	32.1	9.0 E-06
Gallbladder alterations (%)	38.8	57.6	0.0150
*Clinical signs*			
Death in hospital (%)	37.8	17.1	0.0013
Begining of symptoms (days)	89±198	104±257	0.68
Digestive haemorrhages (%)	22.0	36.7	0.0374
Esophageal varices (%)	51.3	82.3	8.8 E-12
Ascites (%)	73.6	82.0	
Encephalopathy(%)	21.7	44.4	0.0841 ns
Pain (%)	72.8	31.7	4.3 E-08
Pain in right quadrant/Epigastralgia (%)	61.7	12.6	3.7 E-12
Nausea (%)	63.1	27.2	0.0183
Fever (%)	15.6	32.5	0.0147
Dizziness (%)	28.1	20.6	ns
Jaundice (%)	5.2	18.0	0.0725 (ns)
Portal thrombosis (%)	51.4	53.1	ns
Hepatomegaly (%)	29.6	9.0	0.0750 (ns)
Splenomegaly (%)	81.1	37.0	2.5 E-09
	70.8	63.8	ns
*Biochemistry*			
AFP (ng/mL)	6765±23000	4±3	0.0783 (ns)
ALP (IU/mL)	242±230	191±303	0.29 (ns)
Urea (mM)	6.5±6.5	5.1±3.4	0.0576 (ns)
AST (IU/mL)	151±178	104±137	0.0384 (ns)
ALT (IU/mL)	72±102	61±92	0.46 (ns)
GGT (IU/mL)	256±238	151±313	0.0201
Total bilirubin (microM)	87±130	66±85	0.20 (ns)
CRP (mg/L)	64±67	32±44	0.0042
Proteins (g/l)	70±18	62±16	0.0574
Creatinine (microM)	94±88	62±16	0.12
Uric acid (microM)	387±256	417±318	0.7133
*Hematology*			
Leukocytes (/mm^3^)	8900±1067	6128±3397	0.0064
Neutrophils (%)	61±15	56±15	0.0251
Lymphocytes (%)	24±13	28±14	0.0991
Monocytes (%)	9±4	11±6	0.0196
Hemoglobin (g/l)	10.3±2.1	9.4±2.5	0.0062
Plaquettes (Giga/L)	207±112	109±86	5.1 E-11
INR	1.6±0.6	2.4±1.2	4.6 E07
*Non Invasive scores for liver function*			
De Ritis ratio	2.3±1.9	2.1±1.6	0.33 (ns)
APRI	2.2±3.3	3.3±4.0	0.0612 (ns)
FIB-4	5.2±4.4	7.7±6.5	0.0043
GPRI	2.4±1.7	3.2±6.1	0.35 (ns)
MELD	13.5±5.8	17.7±6.6	6.5 E-05
APPRI	1.2±1.3	2.2±2.3	0.0063
API	4.9±2.7	6.6±2.1	5.9 E-06
CDS	7.0±2.3	9.1±2.9	8.0 E-07
FibroQ	13.5±16.4	35.8±42.8	1.1 E-04
GUCI	6.5±10.8	14.7±27.3	0.0206
King’s score	79.0±103.5	154.0±228.7	0.0126
*Survival prediction score*			
NLR	4.5±5.9	4.4±13.7	0.99 (ns)
PLR	251±497	235±933	0.89 (ns)
MLR	0.57±0.65	0.94±3.14	0.32 (ns)

AFP, alpha-fetoprotein; ALT, Alanine aminotransferase; ALP, Alkaline phosphatase; APRI, aspartate aminotransferase to platelet ratio index; APPRI, Alkaline Phosphatase to Platelet ratio Index, AST, Aspartate aminotransferase; CRP, c-reactive protein; FIB-4, fibrosis index based on four factors; GGT, Gamma glutamyltransferase; GUCI, Goteborg University cirrhosis index; HBsAg, Hepatitis B surface antigen; HCC, Hepatocellular carcinoma; INR, international normalized ratio; LC, Liver Cirrhosis; MELD, model for end-stage liver disease; MLR, Monocytes-to-Lymphocyte Ratio; NLR, Neutrophil-to-Lymphocyte Ratio; PLR, Platelet-to-Lymphocyte Ratio. na, not available; ns, non-significant.

**Figure 1:**
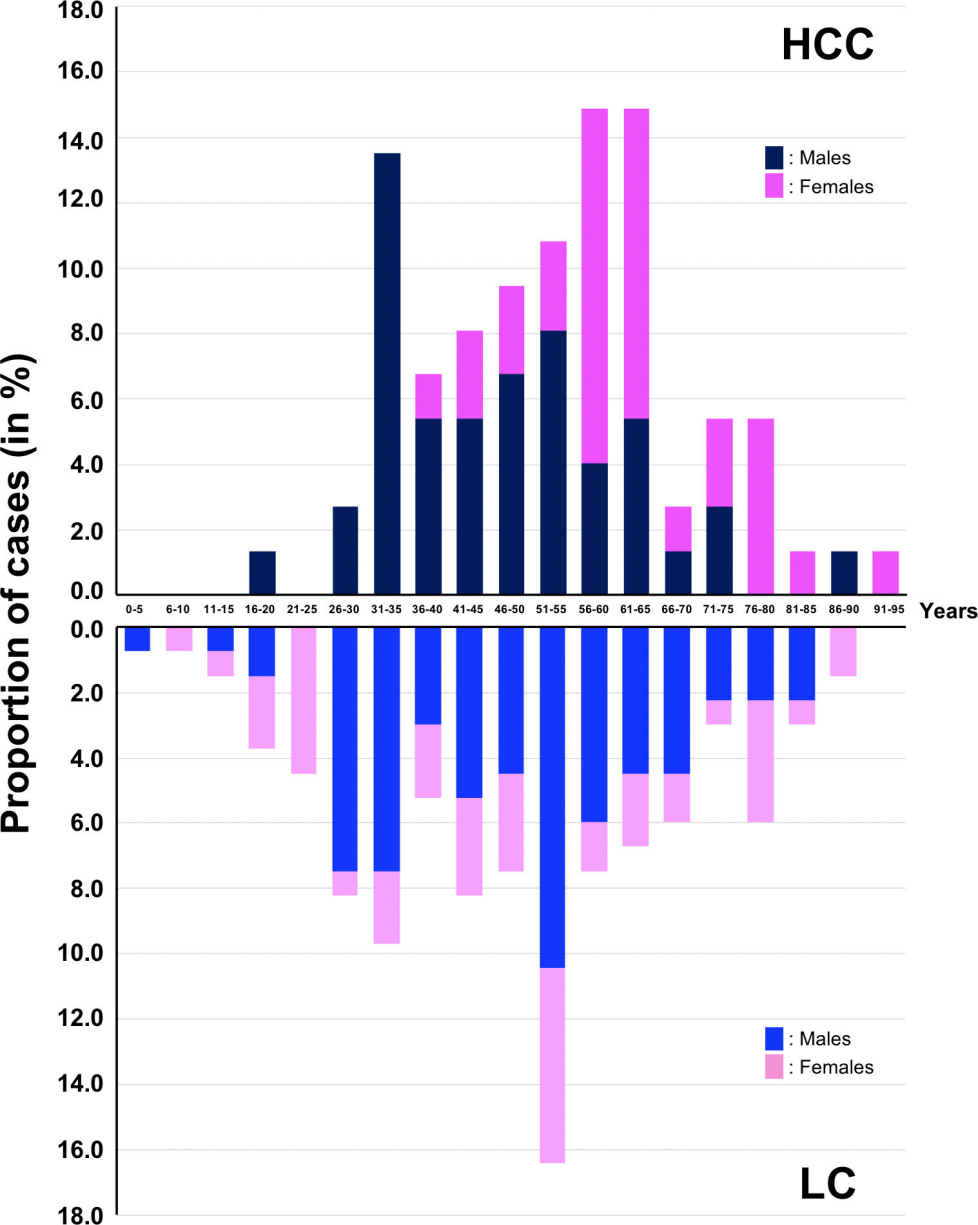
Distribution of HCC and LC cases according to age.

This population was composed of 127 men and 81 women aged from 3–91 years with a mean age 50.3 ± 17.9 years. The mean ages of patients with HCC or LC were non-significantly different with tumor patients in their early sixth decade (53.2 ± 15.7 years) of lifespan, while the cirrhotic patients were a little bit less than 5 years younger (48.6 ± 18.6 years, see **Table 1** and **Figure 1**). The Male : Female sex ratios were low (1.4 in HCC, 1.8 in LC) for both pathologies. Serum AFP was frankly elevated (>400 ng/ml) in only 35.8% of HCC cases.

### Risk factors

Concerning RF, chronic viral infections were the dominant etiologies present in 74.6% and 61.8% of HCC and LC, respectively. In HCC, chronic infection with HBV (seropositivity for HBsAg) was the most frequent (43.4%) followed by anti-HCV reactivity (34.3%). Dual infections were infrequent (3.1%). In LC, corresponding values (35.9% and 28.5%) were slightly lower but not significantly different from HCC. Seropositivity for HIV was significantly more frequent in HCC than in LC (14.5% vs 5.1%, p = 0.015).

The prevalence of toxic RF responsible for severe liver diseases and ethanol and tobacco consumption were not significantly different in HCC than LC (**Table 1**). The same was true for the prevalence of type 2 diabetes (T2D). Overall, a complete absence of RF for liver disease, i.e., a cryptogenic origin (most probably a non-alcoholic fatty liver disease, NAFLD), was rare (6.7% and 7.7%) in both pathologies. It is *a posteriori* plausible to consider that a large subset of these cryptogenic cases is composed of unidentified cases of non-alcoholic fatty liver diseases (NAFLD).

### Clinical features

Tumor characteristics and imaging features clearly indicated that neoplastic processes were overall diagnosed at very advanced stages. The main dimension of the principal tumor mass was 84 ± 50 mm and HCC was already multifocal in almost 73.0% of cases. In HCC patients, liver tissue was cirrhotic in 91.7% of cases with a macronodular pattern in two thirds of the cases, a proportion significantly higher than patients with LC (45.2%, p = 0.010) presumably in keeping with the slightly higher proportion of viral etiologies in HCC.

With regard to clinics, signs of decompensation were present in 98.4% of LC cases and in 88.2% of HCC cases (p = 0.003) indicating that patients with LC, similar to HCC patients, were seeking care at a hospital facility when the disease was already very advanced. According to the patients, symptom onsets were dating back to 89 ± 198 days and 104 ± 257 days earlier for the patients with HCC and LC, respectively.

HCC patients were essentially characterized for their higher prevalence of pain and hepatomegaly, significantly more frequent for obvious reasons (**Table 1**). Features typical of the terminal phase of LC (esophageal varices, digestive bleeding, hepatic encephalopathy) were of course more frequent, sometimes very significantly, in this condition than in HCC (**Table 1**).

### Biological features

Few biochemical variables (AST, g-GT, CRP) were significantly different between HCC and LC patients. Hematological features were almost invariably significantly differing between both conditions with HCC patients displaying clear signs of inflammation (more leukocytes, higher proportion of neutrophils) in blood cell counts while LC patients were displaying consistently worsened values for platelets and coagulation (INR, **Table 1**). As expected, non-invasive scoring systems to estimate liver fibrosis were systematically worse in case of LC.

### Clinical correlations

We next set out to better characterize the differences in presentation installed by the different etiologies responsible for HCC or LC developments in Gabon.

**Figure 2:**
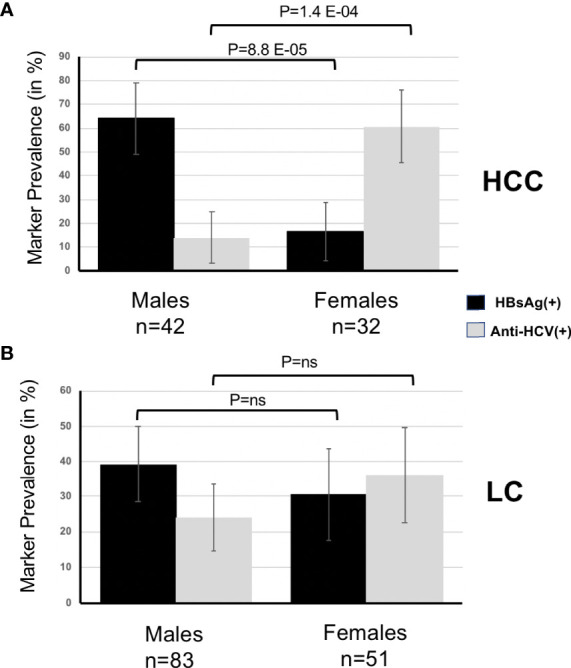
**(A)**, Prevalence of HBsAg and anti-HCV according to sex in HCC. **(B)**, Prevalence of HBsAg and anti-HCV according to sex in LC.

In HCC, the most striking divergence concerned the distribution of viral RF according to sex. Seropositivity for HBsAg was the hallmark of male patients (83.5% vs. 35.9%, OR = 8.6, 95%CI = 2.5–35.5, p = 8.8 E-05) whereas the presence of anti-HCV was primarily associated with females (73.8% vs. 22.7%, OR = 9.1, 95%CI = 2.5–39.9, p = 1.4 E-04). Remarkably, this stringent dichotomy was not present in LC (**Figures 2A, B**).

**Figure 3:**
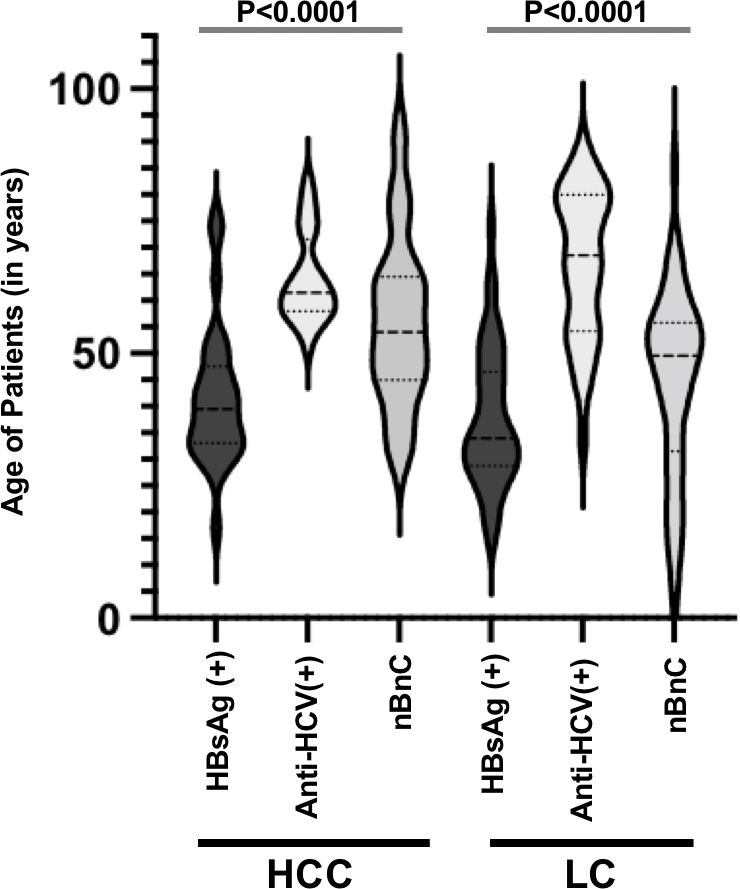
Age of patients in HCC and LC according to the main etiologies.

Age at disease presentation was strongly conditioned by viral etiology as well. In HCC, patients infected with HBV infection were affected with a 2-decade earlier onset than those infected with HCV (42.9 ± 13.9 years vs. 63.8 ± 7.9 years, p = 5.0 E-07). A similar situation was observed in LC (39.4 ± 14.7 years vs. 65.9 ± 14.6, p = 1.1 E-12, **Figure 3**).

**Figure 4:**
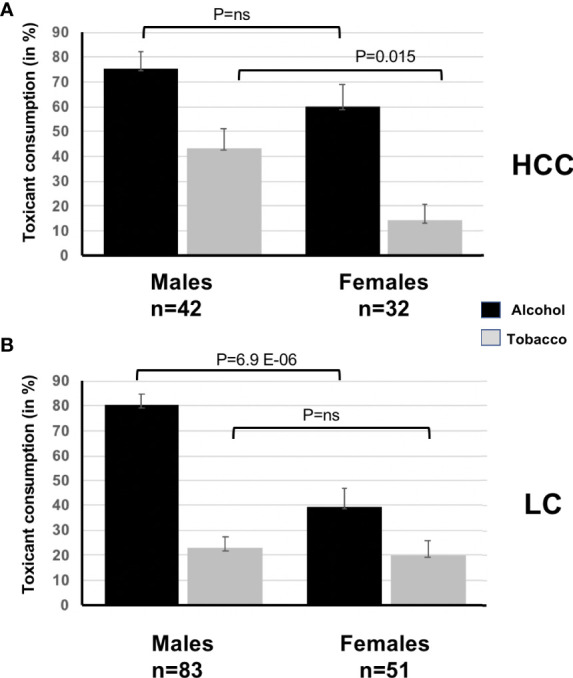
**(A)**, Prevalence of toxic risk factors (ethanol and tobacco) according to sex in HCC. **(B)**, Prevalence of toxic risk factors (ethanol and tobacco) according to sex in LC.

Concerning toxic RF, while ethanol consumption was not significantly different between sexes in HCC, use of tobacco was more frequent in men (45.3% vs. 14.2%, OR = 4.5, 95%CI = 1.2–21.5, p = 0.015). In LC, on the contrary, it was the prevalence of ethanol consumption that made the difference, being almost systematic in men and less frequent in women (83.5% vs. 39.5%, OR = 6.1, 95%CI = 2.6–14.8, p = 6.9 E-06, **Figure 4**). Of course, both for HCC and LC, alcohol and tobacco were often associated. Consequently, an important subset of alcohol consumers were also tobacco users (44.4% vs. 4.5% of drinkers were also smokers, OR = 16.2, 95%CI = 2.2–726.0, p = 6.8 E-04). In LC, corresponding proportions were 29.2% vs. 9.0% (OR = 4.1, 95%CI = 1.3–211.6, p = 0.012).

In HCC, a metabolic RF such as type 2 diabetes (T2D) tended to be more frequent in women (28.0% vs. 10.2%, OR = 3.1, 95%CI = 0.6–16.7, p = 0.099, ns) and more frankly associated with presence of anti-HCV as this virus is a well-known diabetogenic factor (35.0% vs. 5.7%, OR = 8.5, 95%CI = 1.4–94.2, p = 0.008) (11). Likewise, in LC, T2D was also found in a significantly higher proportion of anti-HCV(+) patients (21.2% vs. 3.6%, OR = 6.9, 95%CI = 1.4–44.6, P = 0.005). Because T2D is a non-communicable disease with a maturity onset, its association with anti-HCV (70% of T2D cases) was usually the hallmark of a late HCC onset. T2D was found in HCC patients much older than the non-diabetic ones (66.0 ± 12.8 years vs. 51.0 ± 15.1 years, p = 0.0027). A closely similar situation was also prevailing in LC (60.1 ± 19.4 years vs. 47.6 ± 18.4 years, p = 0.0439).

Alcohol consumption, presumably because of its widespread albeit potentially underestimated prevalence (69.0% in HCC and 65% in LC), was apparently not delineating any specificities in terminal liver diseases of Gabon. The only significant impact of ethanol consumption was its association with smaller tumors (75 ± 50 mm vs. 106 ± 40 mm, p = 0.014) presumably due to its systematic association with advanced fibrosis and its intrinsic toxic effect that enact together and tend to prevent the development of massive tumors.

**Figure 5:**
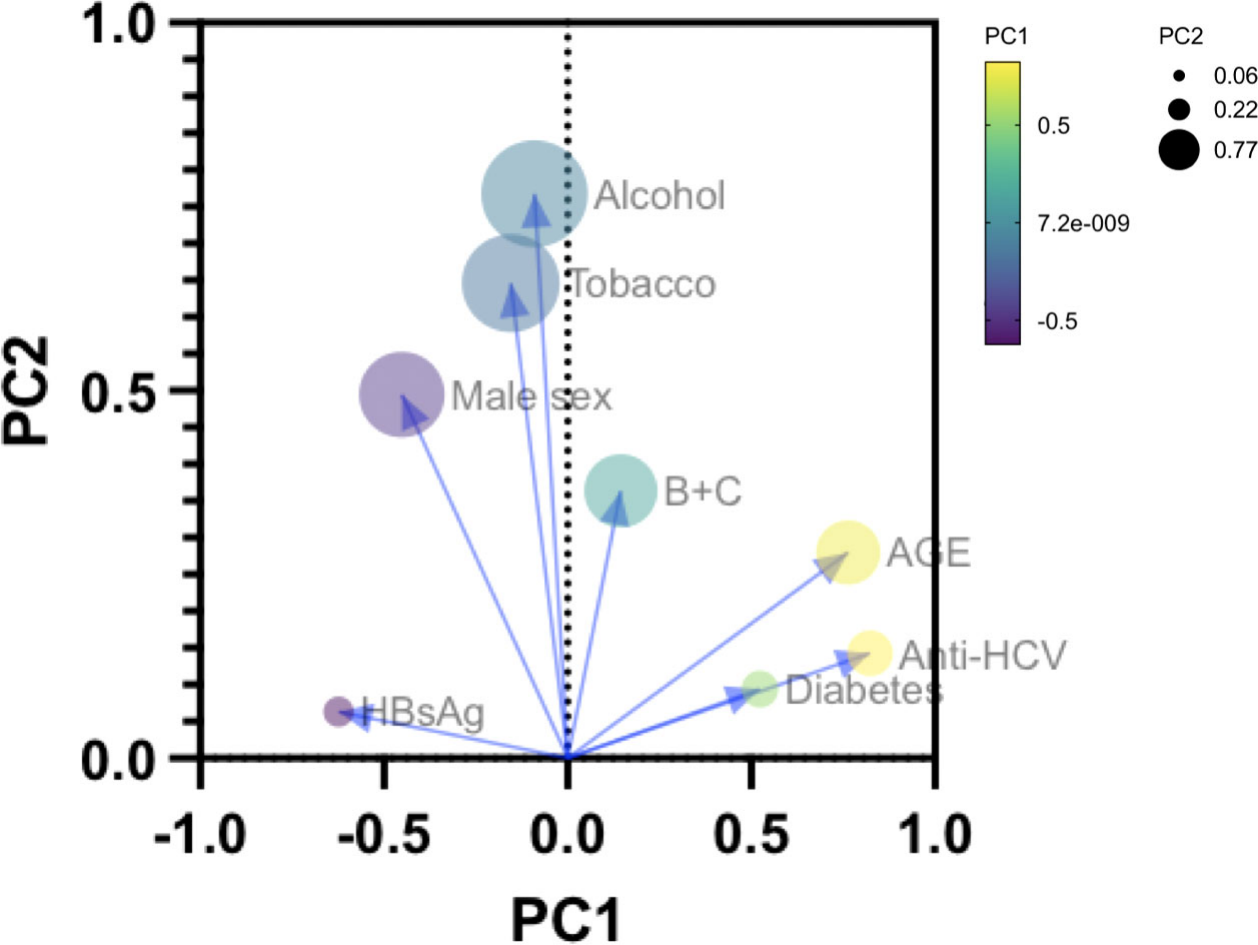
Principal Component Analysis (PCA) describing the interactions of age, sexes, and the principal risk factors (HBsAg, anti-HCV, alcohol consumption, tobacco use and diabetes) of LC (n=134) and HCC (n=74) in Gabon.

To further illustrate the stringent dichotomy of risk factor distribution according to age and sex, we proceeded to a principal component analysis (PCA) regrouping both LC and HCC cases (**Figure 5**). We observed, as indicated by simple statistical tests, that HBsAg, toxicants (alcohol, tobacco), and male sex tend to regroup in the first principal component (PC1) while anti-HCV and age (and diabetes) point in the inverse direction. The PCA suggests that, in Gabon, risk factors for severe liver diseases target gender differentially with a drastic heterogeneity of onset regarding age.

### Clinical biochemistry and hematological alterations

**Figure 6:**
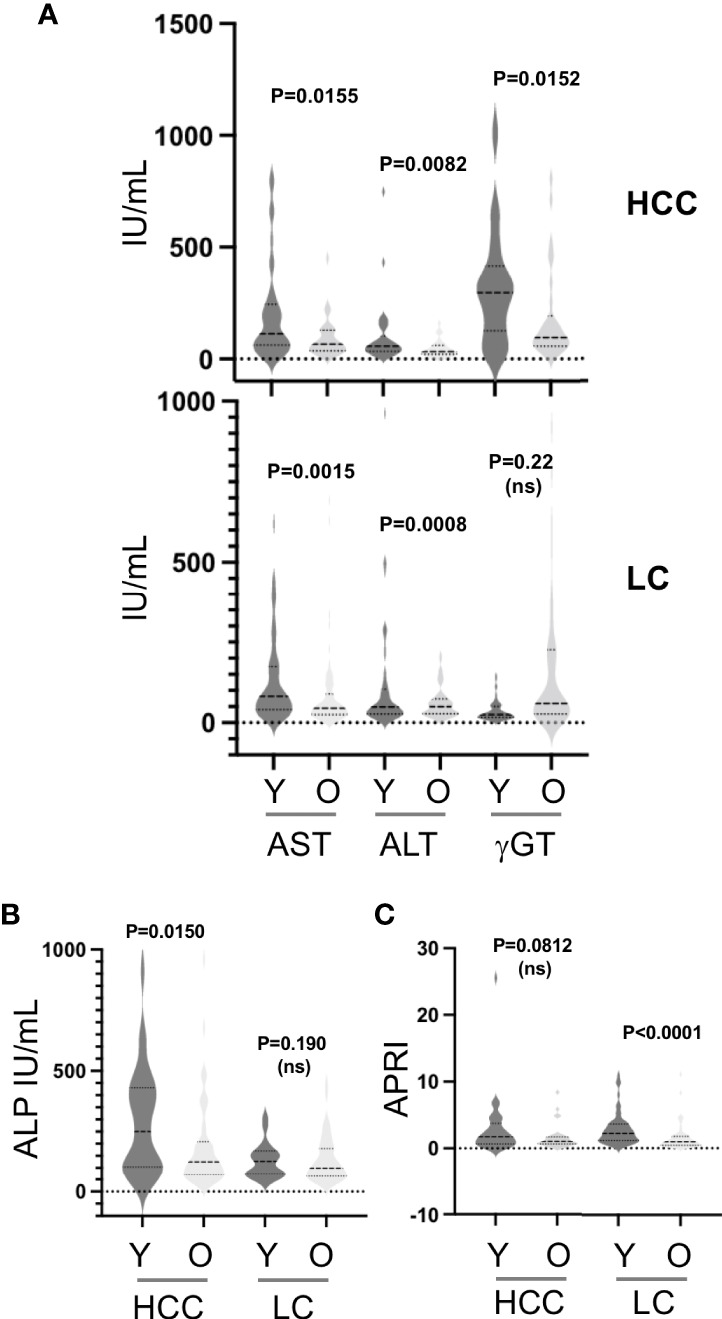
**(A)**, Serum concentrations of aminotransferases (ALT, AST) and gamma-glutamyl-transferase (GGT) in HCC and LC according to the median for age of each group. **(B)**, Serum concentrations of alkaline phosphatases (ALP) in LC and LC according to the median for age. **(C)**, Calculated ALT-to-Platelets Ratio Index (APRI) in HCC and LC according to the median for age.

In HCC, and to a lesser extent in LC as well, young age, a proxy for HBV infection, was generally associated with significant worsening of biological variables. In the younger median for age (Y), liver enzyme values (AST, ALT, g-GT) were significantly higher than in the older median (O, **Figure 6A**). The same was true in HCC only for alkaline phosphatases (ALP, **Figure 6B**), and in LC only, for AST-to-platelet ratio index (APRI, **Figure 6C**). These observations indicate both an ongoing violent liver-damaging process and deep alterations at the time of diagnosis.

Both HCC and LC patients when HBsAg(+) presented additional variables significantly disturbed such as aminotransferases, total bilirubin, APRI, or MELD score (Model for End-Stage Liver Disease, that combines albumin and total bilirubin concentrations, Index Normalized Ratio, and presence of ascites and encephalopathy, not shown), this latter variable assessing disease severity. In comparison, and to the notable exception of renal status markers (urea, creatinine), biological features in anti-HCV(+) HCC patients were much less extensively altered, suggesting a more indolent liver-damaging process.

## Discussion

In Sub-Saharan Africa (SSA) liver cancer is the third highest cancer for men and the sixth for women (Globocan, 2020). There are usually large differences in the prevalence of viral RF between low- and middle-income countries and high-income countries (12). Although this is not systematic, hepatitis viruses largely predominate as causes of liver cancer in low- or middle-income countries (13). The description of the clinical presentation and etiological factors of HCC in SSA is crucial to provide a better appraisal about infectious agents and for the conjecture of their possible transmission routes (14). But a thorough description is also necessary to better understand the influence of other factors such as nutritional transition and exposure to environmental toxins (15, 16).

The present retrospective study aims to contribute to a better understanding of the etiologies of both HCC and LC in Gabon (Middle Africa). It was conducted in the largest hospital in Gabon used as a referral center for digestive diseases.

The demographic characteristics of HCC cases observed in Gabon tend to differ from previous descriptions of the disease in SSA. Regarding the mean age (50.3 ± 15.7 years) of patients, it appears already different from that recently observed in West-African countries such as Ghana (17) and Senegal (18), which is respectively 45.2 and 47.4 years (17. HCC onset in Gabon appears later than what is commonly reported in SSA countries but remains in keeping with descriptions made by Kew and Didi-Kouko *et al.* (19, 20). It is however much earlier than in European countries where it ranges between 63.3 and 70.9 years (21). The mean age of patients with HCC observed in Gabon confirms that the oncogenic process always takes place early among the Sub-Saharan population. The young age of male patients is of course primarily explained by endemicity of some RF like HBV infection transmitted either perinatally from mother to child or horizontally between immune-tolerant children living under the same roof (22). In keeping with the position of Gabon among the low and middle-income countries, the dominant etiologies were viral infections either with HBV or HCV found in 75% and 63% of HCC and LC respectively. However, at variance with a large subset of Sub-Saharan countries, HBV is not a hyper-dominant risk factor of HCC in Gabon as HCV tends to be on par with it, a situation already reported for Senegal (13, 23). This situation indicates that despite the availability of effective interventions for the prevention and treatment of hepatitis B and C, these infections still remain the main causes of HCC and LC burden in Gabon and will continue until the maturity of generations born before 2004 who did not benefit from the universal immunization against HBV at birth (24). In contrast with the situations observed in East European countries as Moldova or in Mongolia, dual infections were infrequent in our study (3.1%) (25, 26). This observation is in keeping with the fact that in Sub-Saharan Africa, HBV and HCV are generally transmitted through different routes and not predominantly together as when intravenous drug use is widespread (27, 28).

Our findings highlight the need for increased efforts in the population screening and the prevention of HBV and HCV transmission to decrease the incidence of severe liver diseases in Gabon. Furthermore, these findings need to be connected with anti-hepatitis B vaccination data. In fact, since 2004, Gabon has adopted vaccination against HBV in the frame of the expanded program of immunization. At weeks 3–4 after birth, all children must receive their first dose of vaccine. According to the 2012 Demographic Health Survey of Gabon, 62% of children (<12 months) born between 2007 and 2012 received their first dose within a month following birth, a proportion that unfortunately let a considerable space to HBV. In addition, a wastage rate of up to 21% from the first to the third dose was also observed (29). Moreover, Gabon did not introduce the anti-HBV birth dose giving time and space to early life contamination. Due to the local epidemiological context, it is essential to implement anti-HBV birth dose immunization (30). Thereby, a decrease of perinatally acquired HBV infections is essential to prevent further progression toward chronicity following first contact with the virus and to drastically reduce HCC and LC incidence 4–5 decades later.

A remarkable feature of our work is the striking divergence concerning the distribution of viral RFs according to sex in HCC. In fact, seropositivity for HBsAg is 3.8 times higher among male patients compared to women, and the presence of anti-HCV is 4.4 times more often associated with women than men. To our knowledge, this is the second observation of such drastic dichotomous distribution in SSA as it was observed 2 decades ago by Kirk and colleagues in Gambia (31). However, such divergent distribution has been recently described in Algeria and Tunisia (32, 33). There is currently no clear explanation for this situation. Of course, it is well-known that little boys get more easily chronically infected with HBV than girls due to an intrinsic susceptibility of the male sex to this infection (34). However, given that transmission routes of HCV necessitate a cutaneous or an epithelial effraction, the excess of HCV infection within female population of HCC cases suggests that Gabonese women might be specifically exposed to this virus. It is, thus, tempting to suspect women-specific iatrogenic routes or esthetic practices (35). Finally, Gabon represents a specific model in SSA. Based on the data of Libreville, drastically different patterns combining sex, viruses, and metabolic and toxic factors prevail and delineate two pathophysiological processes for HCC in Gabon. Male patients are primarily HBV carriers and common users of recreational toxicants such as alcohol and tobacco. By contrast, type 2 diabetes (T2D) tended to be more frequent in women, themselves primarily infected with HCV. This combined dimorphism warrants further studies.

It is also fair to acknowledge that our work presents important shortcomings. The lack of histological or elastometric assessments of non-tumorous and neoplastic tissues is one of them. Another issue is obviously the small sample size that prevents efficient analysis of potentially more complex interactions of risk factors with age and sexes of the patients. A third problem lies in the fact that working retrospectively we did not have the possibility to establish important scores of severity such as Child-Pugh-Turcotte or Barcelona Clinic Liver Cancer stages.

Our observation underlines the necessity for public health authorities to conduct, especially for deadly diseases, RF assessment in local populations in order to implement rapidly information sessions and prevention policies. Moreover, the necessity for Gabonese health stakeholders to elucidate the transmission routes of HCV in women is all the more necessary in that, at the moment, there is a lack of evidence indicating that HCV transmission has been stopped and merely concerns a cohort of women in the seventh decade of their lifespan.

## Conclusion

In conclusion, this study is the first to provide epidemiological description of HCC in Gabon, a country located in Middle Africa. A remarkable feature of our work is the striking sex-dependent distribution of viral, metabolic and toxic risk factors in HCC. Further studies are now warranted to identify routes of HCV transmission and if they are still fueling reservoirs of future patients. Moreover, public policies to prevent alcohol-related harm have also to be urgently implemented in Gabon.

## Data availability statement

The original contributions presented in the study are included in the article/supplementary material. Further inquiries can be directed to the corresponding authors.

## Ethics statement

The studies involving human participants were reviewed and approved by CHUL Institutional Review Board. Written informed consent for participation was not required for this study in accordance with the national legislation and the institutional requirements.

## Author contributions

PP and J-BMK contributed to conception and design of the study. PEIB, PM-B and MS collected data. AM-O, BI and BB-M organized the database. PP performed the statistical analysis. PP, MB-P and PEIB wrote the first draft of the manuscript. All authors contributed to the article and approved the submitted version.

## Funding

During this work, PMB was the recipient of a fellowship from the International Union Against Cancer (UICC, Bourse d’Afrique Francophone, BAF). PP was supported by the French Ligue Nationale contre le Cancer (LNCC, *Equipe labélisée* Anne Dejean).

## Conflict of interest

The authors declare that the research was conducted in the absence of any commercial or financial relationships that could be construed as a potential conflict of interest.

## Publisher’s note

All claims expressed in this article are solely those of the authors and do not necessarily represent those of their affiliated organizations, or those of the publisher, the editors and the reviewers. Any product that may be evaluated in this article, or claim that may be made by its manufacturer, is not guaranteed or endorsed by the publisher.

## References

[1] Bray F Ferlay J Soerjomataram I Siegel R Torre L Jemal A. Global cancer statistics 2018: GLOBOCAN estimates of incidence and mortality worldwide for 36 cancers in 185 countries. CA: Cancer J Clin (2018) 68(6):394–424. doi: 10.3322/caac.21492 30207593

[2] Vento S Dzudor B Cainelli F Tachi K. Liver cirrhosis in sub-Saharan Africa: neglected, yet important. Lancet Glob Health (2018) 6:e10060–1. doi: 10.1016/S2214-109X(18)30344-9 30219314

[3] Moussavou-Boundzanga P Mabika B Bignoumba P Marchio A Mouinga-Ondeme A Kombila J. Underestimation of hepatocellular carcinoma incidence resulting from a competition between modern and traditional medicine: the case of Gabon. J Glob Healh Rep (2020) 4:e2020063. doi: 10.29392/001c.13653

[4] WHO. Global status report on alcohol and health. (Geneva, Switzerland: World Health Organization) (2014).

[5] Shephard G. Risk assessment of aflatoxins in food in Africa. Food Addit Contam Part A Chem Anal Control Expo Risk Assess (2008) 25:1246–56. doi: 10.1080/02652030802036222 18608489

[6] Ge X Zheng L Wang M Du Y Jiang J. Prevalence trends in non-alcoholic fatty liver disease at the global, regional and national levels, 1990–2017: a population-based observational study. BMJ Open Gastroenterol (2020) 10(8):e036663. doi: 10.1136/bmjopen-2019-036663 PMC7402189 32747349

[7] Parkin D. International variation. Oncogene. (2004) 23:6329–40. doi: 10.1038/sj.onc.1207726 15322508

[8] McGlynn K Petrick J London W. Global epidemiology of hepatocellular carcinoma: an emphasis on demographic and regional variability. Clin Liver Dis (2015) 19:223–38. doi: 10.1016/j.cld.2015.01.001 PMC4712629 25921660

[9] Jang H Kim T Burns P Wilson S. Enhancement patterns of hepatocellular carcinoma at contrast-enhanced US: Comparison with histologic differentiation. Radiology. (2007) 244:898–906. doi: 10.1148/radiol.2443061520 17709836

[10] Ayuso C Rimola J Vilana R Burrel M Darnell A García-Criado Á. Diagnosis and staging of hepatocellular carcinoma (HCC): current guidelines. Eur J Radiol (2018) 101:72–81. doi: 10.1016/j.ejrad.2018.01.025 29571804

[11] White DL Ratziu V El-Serag HB. Hepatitis c infection and risk of diabetes: a systematic review and meta-analysis. J Hepatol (2008) 49(5):831–44. doi: 10.1016/j.jhep.2008.08.006 PMC2642971 18814931

[12] Llovet J Kelley R Villanueva A Singal A Pilarsky E Roayaie SL. Hepatocellular carcinoma. Nat Rev Dis Primers (2021) 7:6. doi: 10.1038/s41572-020-00240-3 33479224 PMC13537433

[13] de Martel C Maucort-Boulch D Plummer M Franceschi S. World-wide relative contribution of hepatitis b and c viruses in hepatocellular carcinoma. Hepatology. (2015) 62(4):1190–200. doi: 10.1002/hep.27969 PMC5019261 26146815

[14] Yang JD Mohamed EA Abdel Aziz AO Shousha HI Hasheml MB Nabeel MM. Characteristics, management, and outcomes of patients with hepatocellular carcinoma in Africa: a multicountry observational study from the Africa liver cancer consortium. Lancet Gastroenterol Hepatol (2017) 2:103–11. doi: 10.1016/S2468-1253(16)30161-3 28403980

[15] Steyn N Mchiza Z. Obesity and the nutrition transition in Sub-Saharan Africa. Ann N Y Acad Sci (2014) 1311:88–101. doi: 10.1111/nyas.12433 24725148

[16] Liu Y Wu F. Global burden of aflatoxin-induced hepatocellular carcinoma: A risk assessment. Environ Health Perspect (2010) 118:818–24. doi: 10.1289/ehp.0901388 PMC2898859 20172840

[17] Tachi K Agyei-Nkansah A Archampong T. Hepatocellular carcinoma in Ghana: a retrospective analysis of a tertiary hospital data. Pan Afr Med J (2020) 36:43. doi: 10.11604/pamj.2020.36.43.21085 32774619 PMC7388599

[18] Diallo I Ndiaye B Toure M Sow A Mbengue A Diawara PS. Hepatocellular carcinoma in Senegal: epidemiological, clinical and etiological aspects about 229 cases at hopital principal de Dakar. Pan Afr Med J (2021) 38:99. doi: 10.11604/pamj.2021.38.99.25195 33889265 PMC8035678

[19] Kew M. Epidemiology of hepatocellular carcinoma in sub-Saharan Africa. Ann Hepatol (2013) 12(2):173–82. doi: 10.1016/S1665-2681(19)31354-7 23396727

[20] Didi-Kouko Coulibaly J Yeboua M Kouassi Mbengue A Allah Kouadio E Anzouan-Kacou Kissi H Binan A. Evolution of hepatocellular carcinoma epidemiology in côte d’Ivoire. Bull Cancer (2017) 104:937–45. doi: 10.1016/j.bulcan.2017.09.010 29128083

[21] Capocaccia R Sant M Berrino F Simonetti A Santi V Trevisani F. Hepatocellular carcinoma: Trends of incidence and survival in Europe and the united states at the end of the 20th century. Am J Gastroenterol (2007) 102:1661–70. doi: 10.1111/j.1572-0241.2007.01337.x 17555459

[22] Dakurah OB Tamandjou C Zunza M Preiser W Maponga T. Viral hepatitis associated hepatocellular carcinoma on the African continent, the past, present, and future: a systematic review. BMC Cancer (2021) 21:715. doi: 10.1186/s12885-021-08426-y 34144696 PMC8214285

[23] Raza SA Clifford GM Franceschi S. Worldwide variation in the relative importance of hepatitis b and hepatitis c viruses in hepatocellular carcinoma: a systematic review. Br J Cancer (2007) 96(7):1127–34. doi: 10.1038/sj.bjc.6603649 PMC2360117 17406349

[24] Bisvigou U Kamgaing E Rogombe S Adjaou B Ibinga E Ategbo S. Assessment of vaccination status and booster vaccinations in adolescents attending school in Libreville, Gabon. Pan Afr Med J (2020) 35:74–74. doi: 10.11604/pamj.2020.35.74.20024 PMC7250231 32537077

[25] Tsatsralt-Od B Takahashi M Nishizawa T Endo K Inoue J Okamoto H. High prevalence of dual or triple infection of hepatitis b, c, and delta viruses among patients with chronic liver disease in Mongolia. J Med Virol (2005) 77:491–9. doi: 10.1002/jmv.20482 16254981

[26] Turcanu A Pitel A Dumbrava V Tcaciuc E Donscaia A Peltec A. Profile of hepatocellular carcinoma in the repiublic of Moldova: a first-hand information on its presentation, distribution and etiologies. Rom J Int Med (2018) 57(1):37–46. 10.2478/rjim-2018-0026 30375353

[27] Sonderup M Afihene M Ally R Apica B Awuku Y Cunha L. Hepatitis c in sub-Saharan Africa: the current status and recommendations for achieving elimination by 2030. Lancet Gastroenterol Hepatol (2017) 2:910–9. doi: 10.1016/S2468-1253(17)30249-2 29132760

[28] Pinho-Nascimento C Bratschi M Höfer R Soares C Warryn L Pečerska J. Transmission of hepatitis b and d viruses in an African rural community. Msystems. (2018) 3:e00120–18. doi: 10.1128/mSystems.00120-18 PMC6143728 30246145

[29] International DGdlSDeI. Enquête démographique et de santé du Gabon 2012. Calverton, Maryland, et Libreville, Gabon: DGS et ICF International (2013).

[30] Ekra D Herbinger K Konate S Leblond A Fretz C Cilote V. A non-randomized vaccine effectiveness trial of accelerated infant hepatitis b immunization schedules with a first dose at birth or age 6 weeks in côte d'Ivoire. Vaccine. (2008) 26:2753–61. doi: 10.1016/j.vaccine.2008.03.018 18436354

[31] Kirk G Lesi O Mendy M Akano A Sam O Goedert J. The Gambia liver cancer study: Infection with hepatitis b and c and the risk of hepatocellular carcinoma in West Africa. Hepatology (2004) 39. doi: 10.1002/hep.20027 14752840

[32] Chikhi Y Cheraitia S Gougam RO Lounes F Zemmouchi C Belal N. Wide sexual dimorphism of hepatocellular carcinoma presentation in Algeria. Gastrointestinal Tum (2019) 6. doi: 10.1159/000501453 PMC6873072 31768356

[33] Dhifallah I Khedhiri M Chouikha A Kharroubi G Hammami W Sadraoui A. Hepatitis viruses take advantage of traditional practices to increase the burden of hepatocellular carcinoma in Tunisia. Arch Virol (2020) 165:33–42. doi: 10.1007/s00705-019-04440-5 31630275

[34] Ruggieri A Gagliardi M Anticoli S. Sex-dependent outcome of hepatitis b and c viruses infections: Synergy of sex hormones and immune responses? Front Immunol (2018) 9:2302. doi: 10.3389/fimmu.2018.02302 30349537 PMC6186821

[35] Mariano A Mele A Tosti M Parlato A Gallo G Ragni P. Role of beauty treatment in the spread of parenterally transmitted hepatitis viruses in Italy. J Med Virol (2004) 74:216–20. doi: 10.1002/jmv.20182 15332269

